# Color and Luminance Influence, but Can Not Explain, Binocular Rivalry Onset Bias

**DOI:** 10.1371/journal.pone.0018978

**Published:** 2011-05-04

**Authors:** Jody Stanley, Olivia Carter, Jason Forte

**Affiliations:** Psychological Sciences, University of Melbourne, Parkville, Victoria, Australia; Barrow Neurological Institute, United States of America

## Abstract

When an observer is presented with dissimilar images to the right and left eye, the images will alternate every few seconds in a phenomenon known as binocular rivalry. During sustained viewing, the timing of these switches appears to be unpredictable. Recent research has suggested that the initial ‘onset’ period of rivalry is not random and may be different in its neural mechanism than subsequent dominance periods. It is known that differences in luminance and contrast have a significant influence on the average dominance during sustained rivalry and that perception of luminance can vary between individuals and across the visual field. We therefore investigated whether perception of luminance contrast plays a role in onset rivalry. Observers viewed rival targets of equal brightness for brief presentations in eight locations of the near periphery and reported the color that was first dominant in each location. Results show that minimizing differences in brightness and contrast yields a stronger pattern of onset dominance bias and reveals evidence of monocular dominance. The results suggest that both contrast and monocular dominance play a role in onset dominance, though neither can fully explain the effect.

## Introduction

When the eyes view dissimilar images in the same visual location, one's initial perception is that of a fusion of the two scenes [1]. However, without any change to the images, our perception will rapidly shift to one of binocular rivalry so that what we see alternates between the two ‘rivalling’ monocular percepts. This incongruity between constant physical binocular stimulation and changing conscious perception has been used to study brain processes underlying luminance [2], contrast [3], and motion perception [4]. It has also been used to investigate more complex visual processes such as how and where grouping properties are organized [5], and how visual signals reach conscious awareness [6], [7], [8].

Perceptual switching during binocular rivalry is likely to involve changes in neural activity in multiple brain regions (for reviews see [7], [9]). Reciprocal inhibition between monocular neurons located in the primary visual cortex was initially believed to be the cause of perceptual switching [9]. Subsequent research has revealed that neural activity early in the visual pathway, in the Lateral Geniculate Nucleus [10], [11], as well as activity in late stages of visual processing such as frontal parietal regions [12], [13] are correlated with perceptual switching.

Consistent with the majority of research investigating the neurobiological mechanisms underlying binocular rivalry, all of the computational models of binocular rivalry focus on the perceptual dynamics experienced during prolonged viewing of rivalrous stimuli. Many of these recent models are based on neural adaptation and inhibition at multiple levels of the visual system [14], [15]. There are also models based on Bayesian [16] and related cognitive influences [17], as well as models depending on stochastic or random effects [18], [19], [20].

Within this context of past binocular rivalry research, it is important to consider recent evidence suggesting the initial “onset” period of dominance during binocular rivalry depends on mechanisms that are independent of those that explain sustained binocular rivalry dynamics [21], [22], [23]. To date, the vast majority of neurobiological and computational accounts of binocular rivalry have not distinguished between the initial dominance period and subsequent perceptual transitions. Further confusing the matter, a number of studies have used brief presentation paradigms to draw specific conclusions about the factors effecting sustained rivalry.

Following on from earlier demonstrations that different factors appear to be responsible for determining onset dominance compared to dominance during sustained rivalry, this study aims to identify which factors are in fact involved in determining which perceptual state will dominate with the initial stimulus onset. The mechanisms of onset dominance may be of particular interest as they are likely to play an important role in natural viewing behavior, where visual input is always changing as a result of saccadic eye movements and a constantly shifting visual scene. It is therefore likely that perception of binocular mismatches will be heavily influenced by mechanisms that control the “onset” period of dominance.

A handful of studies have identified some of the stimulus conditions that influence onset dominance differently to sustained dominance. For example, small imbalances in contrast have a much greater effect in determining initial dominance than in influencing subsequent switching in sustained rivalry [23]. Endogenous and exogenous attention may also influence initial dominance more than subsequent alterations [22]. In contrast, differences between images that influence psychological attributes like emotional saliency have less effect on onset dominance than during sustained rivalry [24].

One peculiar property of onset rivalry is that the percept that shows a strong bias to dominate in one location may rarely, if ever, dominate in another location of the visual field. This variability in dominance patterns across visual field location suggests low-level mechanisms may play an important role in determining which percept will achieve dominance first. Evidence has shown strong onset biases across the visual field that were stable for weeks, but which varied in pattern between observers and across the visual field for individual observers [21]. These findings were shown to be different from studies demonstrating slowing and even stabilization of dominance when the images were moved around the visual field or intermittently removed from view [25], [26], [27]. Under such conditions, the image that dominated awareness when the stimulus was removed, was also more likely to dominate awareness when the stimulus was presented again, this was true even if dominance had switched prior to stimulus removal [25]. In contrast, Carter and Cavanagh [21] found strong biases in the dominant percept at stimulus onset, irrespective of which percept had been dominant prior to stimulus removal.

In this initial study observers were shown orthogonally oriented binocular grating targets that sinusoidally varied the intensity of the green phosphor to one eye and the red phosphor in the other. Observers were asked to report the first color that was clearly dominant [21]. Using this paradigm, it was found that when the stimulus presented to the two eyes were photometrically matched (isoluminant), the color that dominated initially was very consistent within a location but varied across the visual field [21]. In an attempt to understand the factors contributing to these strong biases in color dominance, we wanted to determine whether contrast differences in these stimuli may be playing a role.

It is well known that non-color opponent mechanisms may show small responses to photometrically nulled chromatic targets. A number of factors are likely to influence the magnitude of such residual achromatic visual responses to alternating chromatic stimuli. For example, the photopic luminance sensitivity function [28] is based on a standard viewer under standard viewing conditions, and is believed to be dependent on the signals from cone photoreceptors with peak sensitivities in the middle (M cone) and long (L cone) wavelength regions of the visible spectrum. There is, however, considerable variability in individual spectral sensitivity for the same stimulus conditions [29]. The sensitivity is also known to vary across the retina due to spectral variability in the filtering of the eye optics [29] as well as physiological variability in L and M cone signals across the retina [30]. Therefore we might expect alternations in photometrically matched red and green stimuli to cause small photopic responses that vary across the visual field. If we assume that small contrast signals therefore exist for isoluminant red-green binocular rivalry stimuli, this residual contrast response may be reduced by using stimuli with red and green intensities adjusted to minimize the perceived motion of a red-green target at each visual field location for each observer [31].

To determine whether onset dominance is sensitive to these very small luminance contrast signals, we assessed whether there was any difference between the pattern of onset dominance for photometrically matched targets and targets that have been matched for perceived brightness at each location of the visual field.

## Experiment 1

To determine whether luminance or contrast imbalances played a role in the onset biases observed in Carter and Cavanagh's [21] study, we explored the effect of minimizing the brightness and contrast differences between the two targets and across the visual field. This was done by using luminance values for the green target that subjectively matched the luminance of the red target for each observer in each of the eight locations used in the rivalry experiment. Both luminance calibration and rivalry experiments were done with a background equaling the average luminance of the red target.

**Figure 1:**
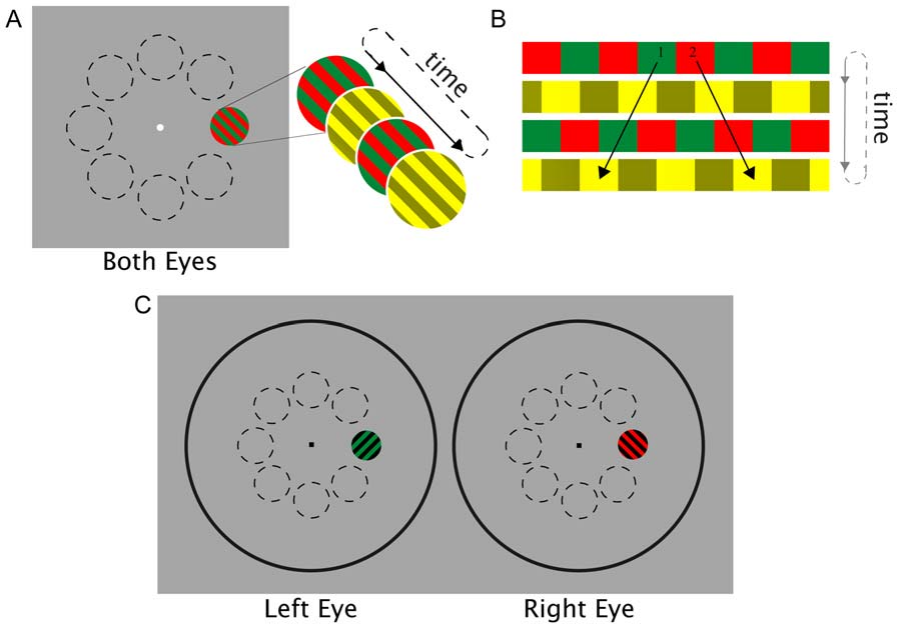
Experiment 1 stimuli. A) Illustration of Anstis and Cavanagh's minimum motion technique that was used to determine equivalent brightness for red and green in each of eight locations (shown with dotted lines). Two square-wave gratings, one of red and green bars and one of light yellow and dark yellow bars, alternate rapidly with each subsequent grating a quarter cycle (half a bar width) offset from the previous one. The gratings were presented for one second at a time. B) The color with greater luminance will seem to track with the lighter yellow bars, moving left if the green bars have greater luminance (1) and right if the red bars have greater luminance (2). C) Examples of stimuli used for rivalry trials: Orthogonal green-and-black and red-and-black gratings overlap to create rivalry in each of the eight outlined locations of the visual field. Using a mirror stereoscope, the left and right circles were overlaid such that the rivalry targets were presented to the same retinal location of the left and right eye respectively. The gray dotted outlines of the eight locations are shown here for the purposes of illustration only and were not part of the experimental display.

To achieve subjectively equal brightness between the red and green targets at each of eight locations of the visual field, we employed a minimum motion technique described by Anstis and Cavanagh [31]. This method utilizes two square-wave gratings, one of red and green bars, and one of light yellow and dark yellow bars. The two gratings alternate at a high frequency with each subsequent grating a quarter cycle (half a bar width) offset from the previous one (see Figure 1A). If red and green values are not perceived to have equal brightness, the brighter color will seem to track with the lighter yellow bars, thus creating the appearance of motion. The bars will appear to move left if the green bars appear brighter and right if the red bars appear brighter (see Figure 1B). When the red and green bars are perceived as being equally bright, neither will appear to have a greater net luminance to pair with the lighter bars, and there will be no apparent motion [31]. The luminance values obtained in the minimum motion trials were then used for the green target for each location in the rivalry trials for each observer (see Figure 1C).

### Materials and Methods

#### Ethics Statement

All participants had normal visual acuity, and gave written consent prior to participation. This study was approved by the University of Melbourne Human Research Ethics Committee In accordance with the Declaration of Helsinki (#0827502.1).

#### Participants

Observers included in this study were two males and three females between ages 21 and 39 years. All were experienced psychophysics observers and included the three authors and two naïve participants.

#### Stimulus

Experimental stimuli were created using an Apple Power Mac G4 computer with MATLAB software using Psychophysics Toolbox routines [32], [33] and presented with a BITS++ Digital Video Processor (Cambridge Research Systems) on a Sony Trinitron Multiscan G520 monitor running at a spatial resolution of 1024×768 pixels with a 120 Hz refresh rate. The luminance versus voltage behavior of the monitor was linearized for each of the red, green and blue phosphors using a Konica Minolta CS-100A colorimeter and gamma corrections to the lookup table.

### Equating Luminance of red and green phosphor across the visual field

Prior to participation in the binocular rivalry portion of the study, luminance values for red and green were equated using the minimum motion technique [31] in each of the eight locations of the visual field that were to be tested. The red bars were presented with full saturation and luminance of 27 cdm^−2^ while the luminance values of the green bars were varied to determine the percentage of full strength green (measured as 77 cdm^−2^) that is perceived as equal to full strength red.

A white fixation point subtending 18 arcmin was located in the center of the screen, with a static gray background of 17 cdm^−2^. The gratings each subtended a visual angle of 2°, had a spatial frequency of 2 cpd, and were located at an eccentricity of 4° from the central fixation point. The minimum motion stimulus cycled at 2 Hz and was presented for 1 sec. This relatively low temporal frequency was chosen to minimize the potential for shifts in equiluminance points that occur at high temporal frequencies [30]. There were a total of 30 trials at each location for each block, and presentations were in random order.

During the minimum motion procedure participants were asked to look through a mirror stereoscope and fixate on the center point as the alternating gratings were presented in each of the eight peripheral locations. Because our targets were viewed binocularly, we can account for variations in isoluminance across the visual field but not between eyes. While it would have been theoretically optimal to also equate the luminance between eyes, this was not technically possible in this experiment - if the red and green targets were presented to separate eyes, any small changes in vergence would put the two targets out of phase and disrupt the minimum motion procedure used here.

The task was two-alternative forced choice with observers asked to indicate, by key press, the perceived direction of motion after each presentation. Each block lasted approximately five minutes and observers completed ten blocks of trials.

**Figure 2:**
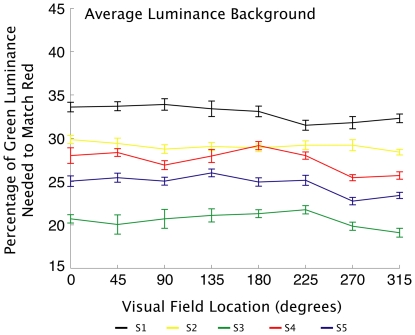
Minimum motion results. Average green luminance values perceived as equal to red luminance in each location for each observer. Green values varied substantially between observers, and also varied somewhat across the visual field of each observer. Minimum motion trials were conducted on a background equaling the average luminance of the red target. Error bars represent standard error.

A QUEST adaptive staircase [34] was used to find the threshold of minimum perceived motion. The adaptive staircase adjusted the luminance of the green value according to the responses of the observer. Values were calculated as the average of the final six reversals of the staircase for each location in each block. The mean of the values found in each of the ten blocks were the final green luminance values used to match the brightness of the patches in the rivalry experiments. Across the five observers and eight locations, there was considerable variation, with green values ranging from 19.1% to 33.9% of full-strength green (see Figure 2). The fact that there were such appreciable differences in luminance detection between individuals and across the visual field of each individual adds further support for the need for luminance matching in each location being tested for onset bias in rivalry if the effects of luminance are to be minimized.

#### Rivalry Experiments

Stimuli for the rivalry experiments were viewed via a mirror stereoscope. During rivalry experiments, a plastic panel with two horizontally arranged circular holes of 4.75 cm radii was placed between the observer and the screen at approximately 23 cm from the observer. Stimuli were viewed through these holes to segregate the right eye stimulus and the left eye stimulus. A chin rest was used so observers could maintain a consistent distance of 57 cm from the screen.

Stimuli for rivalry experiments were presented on the same gray background and in the same locations as those equated for luminance (see Figure 1C). To aid fusion of the left and right eye stimuli, the rivalry stimuli were presented within large circular frames presented to both eyes. Each subtended a viewing angle of 18.5°, with a fixation point subtending 18 arcmin located in the center of each frame. The frame and fixation point were made of black and white noise.

#### Baseline Trials

Prior to conducting trials under experimental conditions, we ran one set of baseline trials with targets matched under similar conditions to Carter and Cavanagh [21]. Green was presented at full saturation (77 cdm^−2^). Red was set at 0.8 saturation (37 cdm^−2^). The grating on each patch had a spatial frequency of 2 cpd, and the respective orientations of the gratings were randomized between red and green targets.

Patches were presented at each location for 1 second in a pseudo-random order (ensuring that within each block there would be exactly 20 trials at each location). Baseline trials were presented in five blocks, with the green target presented to the left eye and the red target to the right eye.

#### Experimental Trials

For all rivalry trials, observers were asked to look through the mirror stereoscope and focus on the central fixation point. After each 1-second rivalry trial, observers were to report, by key press, which color was first dominant. The task was forced choice between the two alternative colors. After each response, the next stimulus patch was presented at another location. The total duration of each block was approximately 2 minutes.

The red target was presented with full saturation and luminance of 27 cdm^−2^. The respective luminance values for the green targets were obtained from the minimum motion procedure described previously. In the first five blocks of trials, as in the baseline trials, the green target was presented to the left eye and the red target to the right eye. During a separate testing session (separated by at least 2 days) a second five blocks of trials were conducted with the green target presented to the right eye, and the red target to the left. Because the minimum motion trials were done binocularly, luminance values obtained for the green target were the same for both conditions. All other aspects of the stimulus and testing procedure were the same as those described for the baseline trials.

### Results

**Figure 3:**
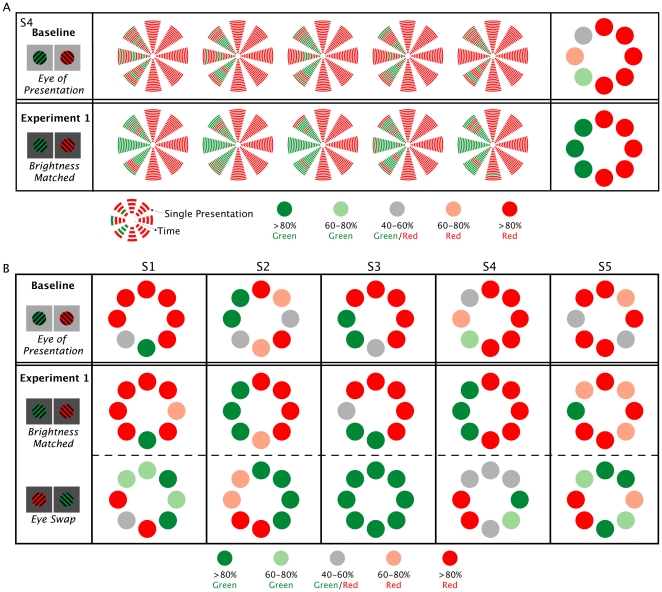
Onset dominance results. A) Representative data is shown for one subject to illustrate perceptual dominance for each stimulus presentation for both baseline and the corresponding eye-of-presentation blocks from Experiment 1 for observer S4. Onset dominance is shown for each 1-second trial in the five testing blocks conducted. Each arc in the eight wedges represents a single presentation in that location, with successive trials represented by an arc one step farther from the center. In the final column, individual presentation data is then averaged, with each circle representing the frequency of the red or green target dominance in each location across the five testing blocks. B) Averaged data shown for all five observers (S1–S5). Baseline: Results of baseline trials show a location-specific bias for red or green targets. Experiment 1: When the brightness of respective green targets were equated to red the onset bias pattern changes and tends to strengthen. When the eye of presentation is reversed, results show reversal in the onset dominance patterns.

Equating for luminance did have a noticeable and consistent effect on the onset dominance pattern in each location (see Figure 3). Contrary to our hypothesis however, reducing the influence of luminance differences did not eliminate the onset bias. Reducing effects of luminance in fact appeared to strengthen the pattern. For most observers, the target that dominated at the onset of rivalry in a given location was less variable than that seen in the baseline trials. Some locations showed a more consistent onset bias than others. Observers typically described dominance in these locations as being more immediate and less ambiguous, with less incidence of incomplete dominance.

Our results indicated that some locations that have a strong bias for one color showed strong bias for the other color when eye of presentation was switched. This pattern of results suggests that there may be differences in the relative strength of the signals coming from each eye. Previous research has demonstrated that such differences in monocular dominance across the visual field do exist and can participate in rivalry during sustained viewing [35]. Carter and Cavanagh [21] did not find any evidence of monocular dominance influence on onset biases when they switched the eye of presentation. In those earlier experiments it is possible, however, that the effects of differences in luminance and contrast may have been so great that any influence of monocular dominance were too subtle to detect. Our results indicate that when imbalances in the perceived brightness and contrast are equated, then clear signs of monocular dominance can be observed in several locations (though individual differences are still considerable). The areas where dominance was strongest after the calibration procedure were the ones that typically showed the most consistent reversal. This suggests that monocular signals are likely to be making a large contribution to the onset bias in those locations.

**Figure 4:**
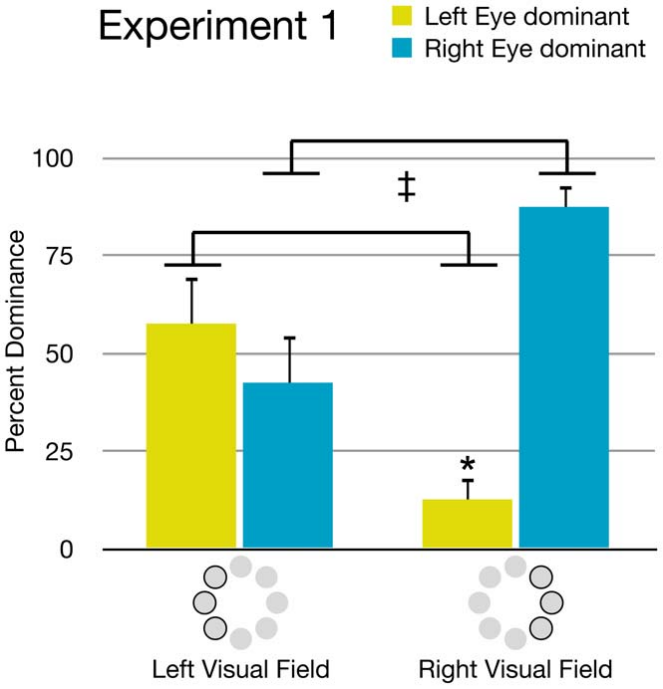
Visual hemi-field effects. Onset bias data from Experiment 1 shows that the left eye's image was significantly more likely to dominate in left visual field locations compared to right visual field locations. In contrast, the right eye's image was more likely to dominate in the right visual field locations compared to left visual field locations (‡ = p<.05). Comparing eye dominance within each of the two hemi-fields, a clear right eye dominance was seen in the right visual hemi-field (* = p<.01). In the left visual hemi-field, the left eye's dominance did not reach significance. Error bars represent standard error.

The distribution of monocular dominance zones was neither total (complete dominance of one eye) nor random. Though there was considerable variation between observers, a few general patterns were apparent (see Figure 4). When targets were presented in the right visual hemi-field, the right eye's target was dominant in an average of 87.4% (SD = 12.1) of presentations, and the left eye's target was only dominant in 12.6% (SD = 12.1) of presentations (p<.01). When targets were presented in the left visual hemi-field there was not a significant difference between the dominance of the right eye's target (M = 42.5, SD = 26.9) and the left eye's target (M = 57.5, SD = 26.9). However, the left eye's target was significantly more likely to be dominant when the targets were presented in the left visual hemi-field than when they were presented in the right hemi-field (p<.05). Likewise, the right eye's target was significantly more likely to be dominant when targets were presented in the right hemi-field than in the left hemi-field (p<.05). These results suggest some bias for targets presented in the temporal visual fields. The greater strength of the right eye's target in the right hemi-field may reflect a combined strength of both right-eye and temporal visual field biases.

## Experiment 2

**Figure 5:**
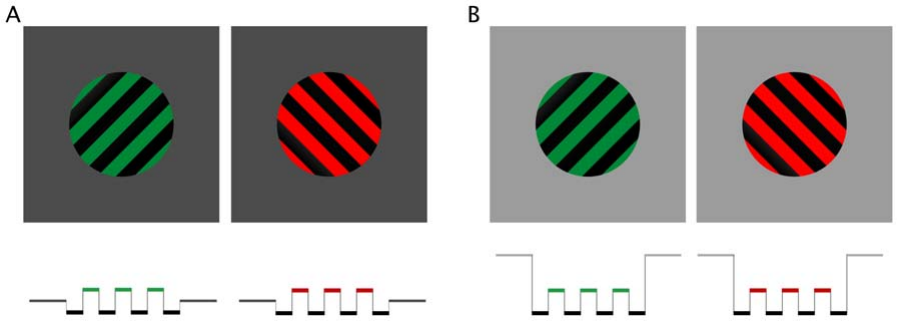
Luminance pedestal. Under each target is a representative luminance profile comparing target luminance to background luminance. A) Luminance profile for Experiment 1: Average luminance of the targets is equal to the luminance of the background. B) Luminance profile for Experiment 2: Average luminance is equal between targets, but the background luminance is higher than average target luminance.

Although the results of Experiment 1 suggest that regional monocular dominance is an important factor in onset bias, not all areas clearly reversed when targets were switched. This result hints that some residual effect of brightness differences may have still been present in the stimuli. In Experiment 1, our aim was to reduce contrast differences across the visual field, providing as few luminance cues as possible. Though the targets were carefully calibrated to be equally bright, slight differences between them may remain. In Experiment 2, we aimed to also reduce any proportional contrast differences between the targets themselves. This was achieved by placing the targets on a luminance pedestal. Under these conditions, the luminance of the background was higher than the average luminance of the gratings (see Figure 5A). Because a brighter background results in greater contrast between the background and the targets, this can be used to reduce the relative impact of any slight luminance differences between the targets.

### Materials and Methods

**Figure 6:**
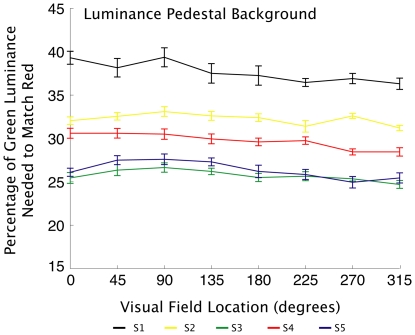
Minimum motion results. Average green luminance values perceived as equal to red luminance in each location for each observer. Minimum motion trials conducted on a background with higher luminance than the average luminance of the targets. For most observers more green luminance is needed to match the red across the visual field when a brighter background is used. Error bars represent standard error.

With the exception of the background luminance, participants, stimuli and procedure were identical to Experiment 1. The luminance of the background was increased to 56 cdm^−2^, and green luminance was calibrated using the minimum motion technique on this background. Results of the second minimum motion calibration again showed considerable variation across subjects and location, with green values ranging from 24.7% to 39.35% of full-strength green (see Figure 6). Relative to the initial calibration procedure described in Experiment 1, when the lighter background was used most observers required more green luminance to match the red across the visual field. The difference between the green values obtained for the two backgrounds demonstrate that the luminance value of the surrounding environment can indeed affect the perceived luminance of the targets themselves.

Rivalry targets were presented as described above, with two sets of five testing blocks. In the first set the red target was presented to the right and the green to the left. In the second set, eye of presentation was reversed.

### Results

**Figure 7:**
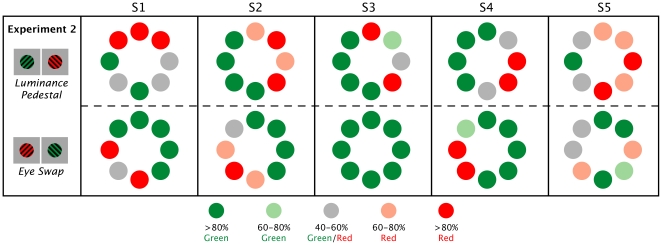
Reported onset dominance. For each of the 5 observer's (S1–S5) each circle represents the average frequency of the red or green target dominance in each location across the five blocks of trials for each observer. Experiment 2: When placed on a brighter background, balanced targets continue to show strong onset bias and evidence of monocular dominance and temporal hemi-field dominance.

**Figure 8:**
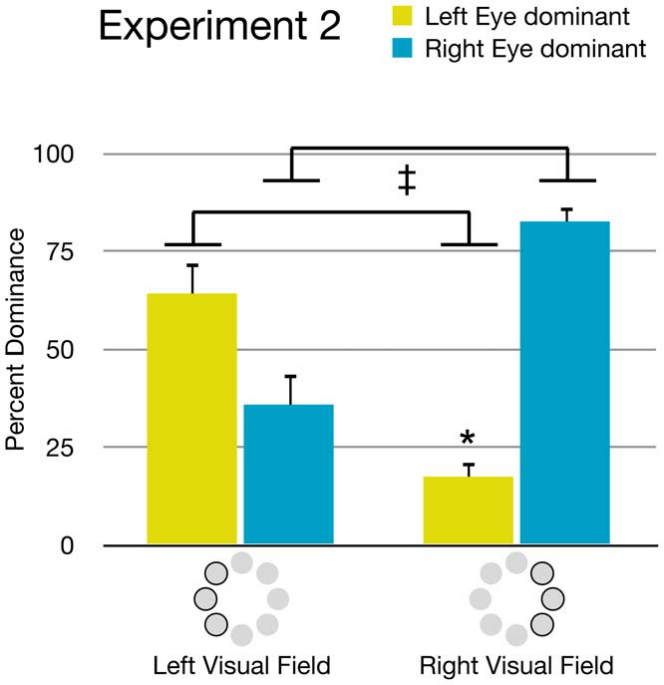
Visual hemi-field effects. Graph showing effects of visual hemi-field on onset bias in Experiment 2. Like Experiment 1, the left eye's image was significantly more likely to dominate in left visual field locations compared to right visual field locations. In contrast, the right eye's image was more likely to dominate in the right visual field locations compared to left visual field locations (‡ = p<.05). Comparing eye dominance within each of the two hemi-fields, a clear right eye dominance was again seen in the right visual hemi-field (* = p<.01). In the left visual hemi-field, the left eye's dominance did not reach significance. Error bars represent standard error.

The high contrast background did result in some changes in the pattern of dominance, though the increased luminance of the background did not eliminate individual differences (see Figure 7). There was still evidence showing regions of monocular dominance. When targets were presented in the right visual hemi-field there continued to be a significant difference (p<.01) between the percentage of right eye dominance (M = 82.6, SD = 8.59) and left eye dominance (M = 17.4, SD = 8.59). In the left hemi-field, no significant difference was found between right eye dominance (M = 35.9, SD = 18.2) and left eye dominance (M = 64.1, SD = 18.2), although the left eye was significantly more likely to dominate when targets were in the left hemi-field than when they were in the right hemi-field, and vice versa (p<.01) (see Figure 8).

**Figure 9:**
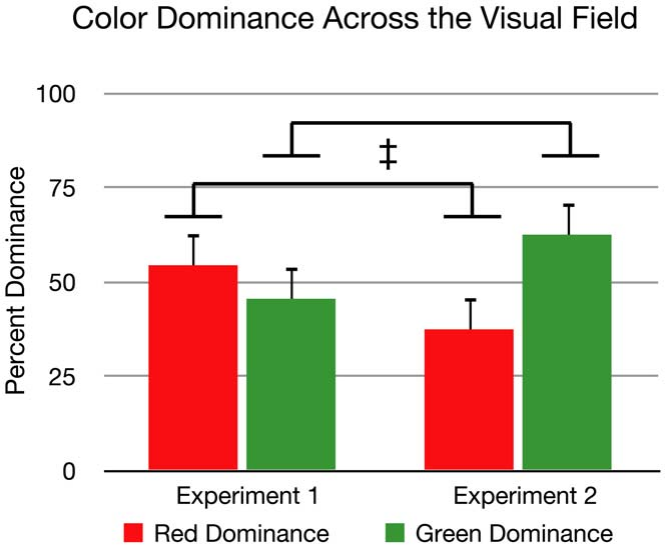
Effect of color. Comparing the percent dominance of each color across the visual field for experiment, there was no significant difference between red and green dominance in Experiment 1. However, there was a trend toward a greater percentage of green dominance compared to red when presented on a brighter background in Experiment 2 (* = p = .097). There was also a significant difference between Experiment 1 and Experiment 2 in the percentages of red and green respectively (‡ = p<.05). Error bars represent standard error.

Interestingly, there was a shift towards green in overall bias compared to Experiment 1 (see Figure 9). Though there was no significant difference between the overall dominance of the red (M = 54.4, SD = 11.9) and green (M = 45.6, SD = 11.9) targets in Experiment 1, in Experiment 2 there was a trend toward a greater percentage of green dominance (M = 62.5, SD = 13) compared to red (M = 37.5, SD = 13) (p = .097). There was also a significant change between Experiments 1 and 2 (p<.05) in the respective amounts of red and green.

## Discussion

Previous studies have demonstrated that the initial period of dominance at the onset of binocular rivalry is likely to be determined by different factors than are responsible for dominance biases during sustained rivalry [21], [22], [24], [36]. In the series of studies reported here, we again found clear localized patterns of dominance at the onset of rivalry. By precisely balancing the perceived brightness of the two rivalry targets in each location for each individual, we were able to show that luminance and contrast do have a considerable effect on the pattern of onset dominance. When the targets were balanced for brightness, the onset bias visibly strengthened. This effect was stable across time within each set of conditions. Though we did not conduct trials of sustained rivalry, Carter and Cavanagh [21] showed that the stable, location-specific dominance biases they found were limited to the onset phase. As our stimuli had even less perceived contrast difference between the targets, we would similarly expect that these factors would have minimal influence on dominance during sustained rivalry.

The current study used Anstis and Cavanagh's [31] minimum motion technique to reduce the effects of luminance that may vary between individuals and across the binocular visual field. While this procedure may not have eliminated all differences in luminance contrast, the fact that clear differences were observed between onset bias generated by photometrically defined stimuli compared to stimuli that were altered to equate for perceived brightness is evidence that such small changes in contrast are relevant to determining which stimulus will dominate at onset.

We found strong and consistent evidence of monocular dominance using our equiluminant displays. While this result is different to those of Carter and Cavanagh [21], they are consistent with an earlier study by Leat and Woodhouse [36]. They showed that flashed stimuli, which involves only the onset phase of rivalry, showed up to 20 times greater sensitivity to ocular dominance than continuously presented rivaling stimuli. In the current study, switching the eye of presentation caused most areas that showed strong onset bias to be dominated by the alternate target. The fact that this eye specific pattern of dominance was not seen in earlier studies using uncalibrated rivalry targets [21] suggests that the influence of monocular dominance may not be sufficient to overcome other imbalances arising from the stimulus or local variations in visual sensitivity.

Interestingly, there was a tendency for the locations on the left and right side of the visual field to be dominated by the stimulus presented to the left and right eyes respectively. This result is in agreement with other studies that have explored the pattern of monocular dominance across the visual field and have shown that temporal visual fields are typically dominant over the nasal visual fields [36], [37], [38]. While evidence of temporal hemi-field dominance is exhibited in our data, our findings are also consistent with Leat and Woodhouse's [36] observation that there is substantial individual difference in the pattern of ocular dominance.

While the majority of research into binocular rivalry has focused on the mechanisms responsible for perceptual awareness and suppression during sustained viewing, a number of recent studies have looked at the effect of perceptual memory and the experience of perceptual stability that can be achieved by brief intermittent presentations (for review see [39]). It should, therefore, be stressed that the onset biases reported here cannot be explained by perceptual memory. This is best illustrated by the detailed individual data shown Figure 3A. It can be seen in this figure that clear biases in the dominant color exist at each location and it is not simply a case of a slowing down of rivalry transitions. Indeed there were instances in which the “non-predominant” color was perceived first, however, this switch to the alternate color was never “stabilized” but rather quickly reverted back to the predominant color in that location.

It is theoretically possible that memory from the preceding trial in alternate location could have had some influence on onset dominance. However, because the transfer of perceptual memory is limited to only small distances [40], any influence of the previously dominant state in the current experiments would be limited to rare cases in which the stimulus was presented to adjacent locations in consecutive trials. Given that there were many examples of adjacent locations showing opposite onset biases it is clear that perceptual memory from the same or adjacent locations cannot explain the biases observed in this study. The question remains open, however, whether perceptual memory was able to play a very minor role in determining perceptual dominance on some trials.

Beyond the effects of perceptual memory, other models interested in the initial period of dominance generally depend on a prior history of unequal binocular stimulation (for example see [40]). In contrast, our paradigm involves a long period of adaptation (approximately 10 sec) to the background adapting field prior to each stimulus presentation. Any inequalities arising from adaptation to the previous 1-second presentation are unlikely to have much of an effect on subsequent presentations. Furthermore, if any residual effect of adaptation to previous stimuli did exist, they should be equally relevant in each location and cannot account for the variability in onset dominance observed across the visual field.

We are not aware of any models that directly discuss the selection of dominance at onset, distinct from the effect of perceptual memory or priming from previous stimuli, in a manner that can account for the findings observed here. There are models concerned more generally with sustained binocular rivalry and in these models there is an acknowledgment that the rivalry process must begin with one stimulus gaining initial dominance over the other. Some models implicate random or stochastic processes [18], [19], [20], however, such random influences are not able to explain the stability of the biases demonstrated here. Similarly, the models depending on Bayesian or cognitive factors [16], [17] are unable to explain the variation between visual field location and the sensitivity to our relatively subtle changes in the stimulus properties observed in our experiments.

It is clear that the onset phase of rivalry is sensitive to factors that vary between individuals and between regions of the visual field within an individual. For a model of onset rivalry to be consistent with the results shown here, it would, therefore, need to contain explicit representation of physiological differences for each observer. After the initial onset phase, more powerful mechanisms may control the alternations in perceptual dominance experienced during sustained rivalry. However, it appears that such mechanisms are either immune to the differences in physiology underlying the onset biases or are able to take them into account.

While further research is required to identify the mechanisms underlying this process, the strength of the onset biases observed implies a winner-takes-all competitive process. Our results support such a race model in which signals originating from respective locations in each eye are sufficiently different in strength to cause slight variation in signal latency (either in terms of the signal being received or achieving a requisite threshold of activation). Only if the signals are truly balanced will stochastic or random fluctuations in neural processing become relevant. Determining exactly where in the brain or which neurons are involved in adjudicating the “winner” obviously requires experiments directed specifically at this question. Irrespective of where or how the initial dominance is resolved, one important implication of such a race model is that it may be the case that only once both stimuli have “arrived” or reached “threshold” that the competitive interactions driving sustained rivalry can begin. Once such interactions are established, the factors determining the initial winner of the race may no longer be relevant – until this point they may be the only factors that are relevant.

### Conclusion

Exploring onset rivalry as a distinct stage in the process of visual competition is important in understanding how the visual system integrates the information received from the two eyes in normal daily vision. As we navigate through the environment, the visual scene that the eyes detect is constantly changing, either from normal saccadic eye movements or from the movement of oneself and other objects in the surround. It is not unusual for an object or surface to cause brief monocular occlusion resulting in the two eyes receiving completely incompatible images. Therefore, the brief presentation of onset rivalry is highly relevant in studying how the brain interprets the rapidly changing, and often inconsistent, signals that are continually received from the eyes. The current study shows that relatively minor differences in the strength of the signal coming from the two eyes—such as those resulting from small contrast imbalances or differences in monocular representation across the visual field—can determine perceptual dominance in a winner-takes-all style of competition. Unlike sustained rivalry, the weaker stimulus may never achieve perceptual dominance during the onset period of rivalry.
